# Does fluctuating selection maintain variation in nest defense behavior in Arctic peregrine falcons (*Falco peregrinus tundrius*)?

**DOI:** 10.1002/ece3.9284

**Published:** 2022-09-13

**Authors:** Nick A. Gulotta, Kimberley J. Mathot

**Affiliations:** ^1^ Department of Biological Sciences University of Alberta Edmonton Alberta Canada; ^2^ Nunavut Wildlife Cooperative Research Unit University of Alberta Edmonton Alberta Canada; ^3^ Present address: Warnell School of Forestry and Natural Resources University of Georgia Athens Georgia USA

**Keywords:** animal personality, assortative mating, behavioral plasticity, selection, state‐dependent behavior

## Abstract

Behavioral expression can vary both within‐ (i.e., plasticity) and among‐individuals (i.e., animal personality), and understanding the causes and consequences of variation at each of these levels is a major area of investigation in contemporary behavioral ecology. Here, we studied sources of variation in both plasticity and personality in nest defense behavior in Arctic peregrine falcons (*Falco peregrinus tundrius*) in two consecutive years. We found that peregrines adjusted their nest defense in response to nesting stage and year, revealing plastic, state‐dependent, adjustment of nest defense. At the same time, nest defense behavior was repeatable in peregrine falcons both within and between years. We tested if fluctuating selection on behavioral types (i.e., individuals average phenotypic expression) and/or assortative mating acted to maintain long‐term among‐individual differences in nest defense behavior. We found that selection on female nest defense differed across years; being positive in 1 year and negative in the other. We also found support for assortative mating in the first year, but disassortative mating in the second. We propose two potential explanations for the observed year‐specific patterns of nonrandom mating: (1) year‐specific plastic adjustment of nest defense and/or (2) changes in the age‐structure of the breeding population. These posthoc explanations are speculative, and require further study. Unfortunately, we could not evaluate this directly with the available data, and future studies are needed with more than 2 years of data on nest‐defense and fitness outcomes, and with a larger number of marked individuals, to properly evaluate these potential explanations.

## Graphical abstract

We studied nest defense behavior in peregrine falcons in two successive breeding seasons to evaluate (1) the extent of repeatable variation in nest defense, and (2) evaluate alternative mechanisms that could maintain this variation. We found that nest defense was repeatable both within‐ and across years, and that patterns of (dis‐)assortative mating and selection on nest defense differed across the two study years. We suggest that changing selection on nest defense leads to plastic adjustment of nest defense behavior that generates changing patterns of assortative mating.

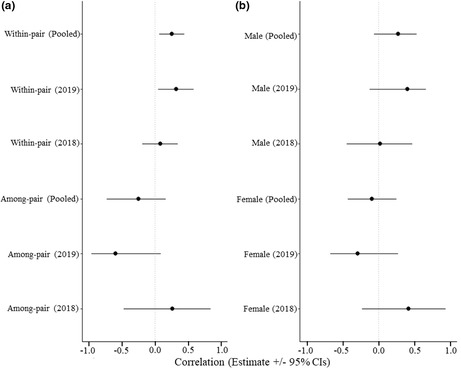

## 1 INTRODUCTION

Within populations, individuals often exhibit consistent among‐individual differences in behavior (i.e., animal personality). Animal personality has been documented in a wide range of taxa and for a wide range of behaviors (reviewed in Bell et al., 2009). Understanding the causes (Wolf et al., 2007; Wolf & McNamara, 2012; Wolf & Weissing, 2010) and consequences (Haave‐Audet et al., 2022; Moiron et al., 2020; Wolf & Kraus, 2014; Wolf & Weissing, 2012) of consistent among‐individual differences in behavior has garnered significant attention over the past two decades. Among‐individual differences in some traits has been shown to have important fitness consequences (Haave‐Audet et al., 2022; Moiron et al., 2020). For example, in Ural owls (*Strix uralensis*), females that are more aggressive in nest defense have higher reproductive success than those that are less aggressive (Kontiainen et al., 2009). This is thought to be due to their greater ability to defend their young from nest predators and/or because parental aggression confers a competitive advantage to offspring when establishing their own territories if those offspring are more likely to also be aggressive (Kontiainen et al., 2009). Several studies examining nest defense have revealed that individuals differ consistently in how they invest in this form of parental care (Arroyo et al., 2017; Both et al., 2005; Burtka & Grindstaff, 2015; Clermont et al., 2019; Dingemanse et al., 2004; Kontiainen et al., 2009). Given that predation of eggs and nestlings is a main contributor to nest failure in many bird species (Montgomerie & Weatherhead, 1988), how can we understand the maintenance of consistent among‐individual differences in nest defense?

Four major (nonexclusive) classes of explanation have been proposed: trade‐offs (Stearns, 1989), state‐dependent behavior (Dingemanse & Wolf, 2010; Wolf & Weissing, 2010), assortative mating (Schuett et al., 2010), and fluctuating selection (Dingemanse et al., 2004). In this study, we assess support for the latter three (state‐dependent behavior, assortative mating, and fluctuating selection) in maintaining consistent among‐individual differences in nest defense in Arctic breeding peregrine falcons (*Falco peregrinus tundrius*) in two successive breeding seasons. Investment in nest defense is commonly thought to trade‐off against investment in provisioning (e.g., Mutzel, Blom, et al., 2013). However, we did not assess evidence for trade‐offs in this study because to do so would have required individual‐level data on provisioning behavior, which we did not have (see Methods and Discussion).

We evaluated support for state‐dependent nest defense in response to a state variable that varied within individuals (i.e., nest stage; also called a labile state variable), and a variable that did not very within individuals (i.e., sex; also called a stable state variable). Many adaptive explanations for consistent among‐individual differences in behavior, including nest defense, are based on state‐dependent behavior. Individual differences in states, including age, nest site/territory quality, energy reserves, and brood value, can lead to individual differences in the expression of behaviors whose payoffs vary with these measures of state (Dingemanse & Wolf, 2010; Wolf & Weissing, 2010). Differences in state that are stable (i.e., not easily changed or not able to be changed) such as sex, offer a simple explanation for repeatable among‐individual differences since the state variable underlying the variation is consistent through time (Dingemanse & Wolf, 2010; Wolf & Weissing, 2010). However, nest defense may also be influenced by labile state variables (i.e., states that can vary within the lifetime of the individual). For example, brood value can vary both within‐ and across‐seasons, and several studies have shown that nest defense increases with increasing brood value (e.g., number or age of offspring; Curio, 1987; Montgomerie & Weatherhead, 1988; Svagelj et al., 2012). These patterns of increasing nest defense as a function of brood value are consistent with parental investment theory, which predicts that increasing brood value should favor more investment into nest defense (Montgomerie & Weatherhead, 1988; Trivers, 1972).

Among‐individual differences in nest defense could also be maintained through nonrandom mating, whereby the fitness of particular combinations of parent behavioral types (i.e., individuals mean phenotypic value for a behavioral trait) have higher success because they are behaviorally and/or genetically more compatible (Class & Dingemanse, 2022; Jiang et al., 2013; Tregenza & Wedell, 2000). Positive assortative mating (hereafter referred to as “assortative mating”) based on behavioral type can contribute to the maintenance of among‐individual differences if pairing with individuals of similar phenotype (i.e., high aggression with high aggression, low aggression with low aggression) has a net positive fitness outcome (Jiang et al., 2013; Schuett et al., 2010; Tregenza & Wedell, 2000). In contrast, among‐individual variation in nest defense could also persist through negative assortative mating (hereafter referred to as “disassortative mating”), whereby individuals prefer a mate that has a dissimilar behavioral type (Jiang et al., 2013; Schuett et al., 2010). However, disassortative mating will erode additive genetic variance (and consequently among‐individual variation) over time if behavioral variation has genetic underpinnings. Thus, negative assortative mating can only explain the long‐term, multi‐generational, maintenance of behavioral variation that arises via (permanent) environmental effects.

Finally, different behavioral types could be maintained in populations under fluctuating selection such that alternative behavioral types achieve equal fitness on average (Dingemanse et al., 2004). Studies have found food availability (Boon et al., 2007; Dingemanse et al., 2004; Montiglio et al., 2013) and population density (Nicolaus et al., 2016) can be key factors underlying fluctuating selection. Fluctuating selection is an intuitive explanation for the maintenance of behavioral types since many taxa experience temporal variations in their environment that directly impact food availability or population density thus allowing multiple optimal behavioral types to exist that are adapted for different ecological contexts, each achieving equal fitness on average.

Here, we study nest defense in Arctic peregrine falcons (*Falco peregrinus tundrius*) in Rankin Inlet, Nunavut, Canada. First, we assess the short (within‐year) and long‐term (across‐year) repeatability of nest defense. We evaluate support for state‐dependence of nest defense by quantifying the relative importance of stable (i.e., sex) and labile (i.e., nest stage) states on the expression of nest defense behavior. Next, we used multivariate models to evaluate support for (dis‐)assortative mating and evaluated whether among‐individual differences in nest defense predicted fitness. To do this, we used the number of young fledged as a fitness proxy, and calculated selection gradients for each combination of sex and year of the study. This allowed us to assess whether there was evidence for fluctuating selection across the two study years, and whether selection differed between the sexes. We discuss our results in light of how they contribute to our understanding of the role of state‐dependence, assortative mating, and fluctuating selection in maintaining variation in nest defense behavior in peregrine falcons.

## 2 METHODS

### 2.1 Study species

Arctic peregrine falcons are long‐distance migrants that winter in the southern United States, Mexico, and Central and South America, and breed throughout the North American Arctic, including Greenland (White et al., 2013). Peregrines arrive at our study site in Rankin Inlet, Nunavut, in mid‐May and egg laying occurs during the first 2 weeks of June (Court et al., 1988). Peregrines do not build nests. Though they will occasionally re‐use Common raven (*Corvus corax*) and Rough‐legged hawk (*Buteo lagopus*) stick nests, most peregrines in our study area nest in scrapes directly on the substrate. Arctic peregrines generally lay between three and four eggs (Court et al., 1988). The incubation period lasts approximately 36 days from when the first egg is laid (33 days from the fourth; Jaffré et al., 2015), and hatching occurs asynchronously in the first 2 weeks of July (Court et al., 1988). Peregrines are long‐lived and exhibit bi‐parental care (Court et al., 1988; Franke et al., 2010). During the breeding season, raptors are central place foragers, and at least one adult is constrained to the nest for incubation, brooding, feeding, and defense of eggs and altricial young (Sonerud, 1992). In peregrine falcons, these behaviors are generally performed by females, which are approximately a third larger than males, and males are commonly engaged in foraging (White et al., 2002). Male peregrine falcons normally provision the female during incubation and early brood rearing (White et al., 2002). Both males and females engage in territorial behavior and defend eggs and young against typical intruders, such as Arctic foxes (*Vulpes lagopus*), Wolverines (*Gulo gulo*), and Short‐tailed weasels (*Mustela erminea*) in our study population (White et al., 2002).

### 2.2 Study site

This study was conducted in a 455 km^2^ area near the community of Rankin Inlet, Nunavut, Canada (62°49′ N, 92°05′ W), situated on the west coast of Hudson Bay. Part of the study area is encompassed within Hudson Bay which is dominated by a rugged coastline that provides suitable habitat for cliff‐nesting species such as peregrine falcons, Rough‐legged hawks, Canada goose (*Branta canadensis*), Common eider (*Somateria mollissima*), and Common ravens. Most of the study area is characterized by rolling upland hills and eskers that contain rugged rocky outcrops that are suitable for nesting (Court et al., 1988). The rugged terrain near the coast, coastal lowlands, and numerous lakes, supports large numbers of Arctic ground squirrels (*Urocitellus parryii*), waterfowl, passerines, shorebirds, and seabird colonies (Court et al., 1988; Hawkshaw et al., 2021a, 2021b).

Arctic peregrine falcons breeding in Rankin Inlet, Nunavut, Canada have been studied since the 1980s to understand their ecology and ecotoxicology following the widespread decline of peregrines from DDT (Court et al., 1988; Franke et al., 2010). Rankin Inlet has one of the highest breeding densities of peregrine falcons in the world with ~30 breeding pairs (i.e., one pair/15 km^2^), believed to be due to the high availability of suitable nesting sites (Court et al., 1988; Franke et al., 2010). This population consumes both mammalian (e.g., Red‐backed vole (*Myodes rutilus*), Northern collared lemming (*Dicrostonyx groenlandicus*), North American brown lemming (*Lemmus trimucronatus*), and Arctic ground squirrels) and avian prey (e.g., insectivorous birds, waterfowl, seabirds; Franke et al., 2010).

### 2.3 Fieldwork

Fieldwork began in mid‐May for both 2018 and 2019 seasons and corresponded with the arrival of peregrines to Rankin Inlet from their annual migration from their southerly wintering grounds. A census of 125 known peregrine nesting sites was conducted at least twice per field season. Sites were considered occupied if one or more adults displayed territorial or reproductive behavior (e.g., vocalization and/or flight behavior associated with defense of breeding territory or presence of nest building, nest, or eggs; following Franke et al., 2010). All unoccupied sites were checked until occupancy was confirmed or the breeding season was sufficiently advanced to conclude that the site was vacant (i.e., mid‐July; Franke et al., 2010). Once eggs were detected, we deployed RECONYX motion‐activated cameras (Recoynyx, Holmen, WI, USA) at the nesting site (i.e., 2018: *n* = 28, 2019: *n* = 34). Occupied nests were visited every ~10 days to replace batteries and memory cards for the duration of the breeding season or until the nest had either failed or fledged. Motion‐activated cameras were used to document prey deliveries, clutch sizes, hatch dates, nest failures, and to read color bands on adults. Once nestlings hatched, a nontoxic colored mark was applied to one leg to track growth until fledging (Anctil et al., 2014; Court et al., 1988). We tracked growth by weighing nestlings at each nest visit using an electronic scale (see Robinson et al., 2017). On the final nest visit, nestlings were sexed and banded (i.e., ~20 days old), and were fitted with a U.S. Fish and Wildlife band (Court et al., 1988; Franke et al., 2010). We did not visit nests after ~25 days to avoid inducing early fledging.

### 2.4 Nest defense scoring

Nest defense was assessed as part of regular nest monitoring (i.e., camera deployment and maintenance, and banding), and occurred during egg laying, incubation, and provisioning stages of the nesting cycle. For each visit, we recorded nest site ID, date, observer, travel mode (i.e., snowmobile, quad, or boat), nest approach direction (from above or below), and time. We did not assess nest defense when traveling by helicopter to remote nest sites since the disturbance associated with this mode of nest approach was markedly different from the other modes (in terms of both noise and visibility, NAG personal observation) which could reasonably be expected to impact nest defense behavior. When observers arrived at the nest, they completed a 2‐min focal observation and recorded several traits that were expected a priori to be indicative of nest defense behavior: flight initiation distance (FID), minimum distance to observer, and number of stoops. Flight initiation distance was the distance, in meters, at which a peregrine flushed from the nest site as the observer approached the nest site. Because peregrine are aerial defenders (White et al., 2002), flushing earlier would be indicative of higher nest defense. Parents that were not at the nest when observers arrived (e.g., were first observed already in flight), were not assigned an FID. When a peregrine was present at some point during an observation session, the minimum distance to the observer was scored. Minimum distance to the observer was recorded at distances between 0 m (i.e., hitting the observer) and 100 m. Individuals observed at distances >100 m were not considered to have exhibited nest defense as this was considered to be too far for meaningful nest defense. Similar thresholds have been used previously in studies of raptor nest defense (e.g., Andersen, 1990). Stoops were characterized by rapid directed movement toward the observer. Closer approaches to the observer and/or more direct attacks on the observer were each assumed to reflect higher nest defensiveness.

When conducting nest defense observations, two observers were present at the nest, one to monitor each parent. Assignment of observers to parents was done arbitrarily for each observation session. Nest defense scoring was adapted from previous studies in raptors (Andersen, 1990; Carrillo & Gonzalez‐Davila, 2013; Wiklund, 1990). Observations of peregrines outside the 2‐min focal observation were not counted toward nest‐defense scores.

### 2.5 Statistical analysis

**FIGURE 1:**
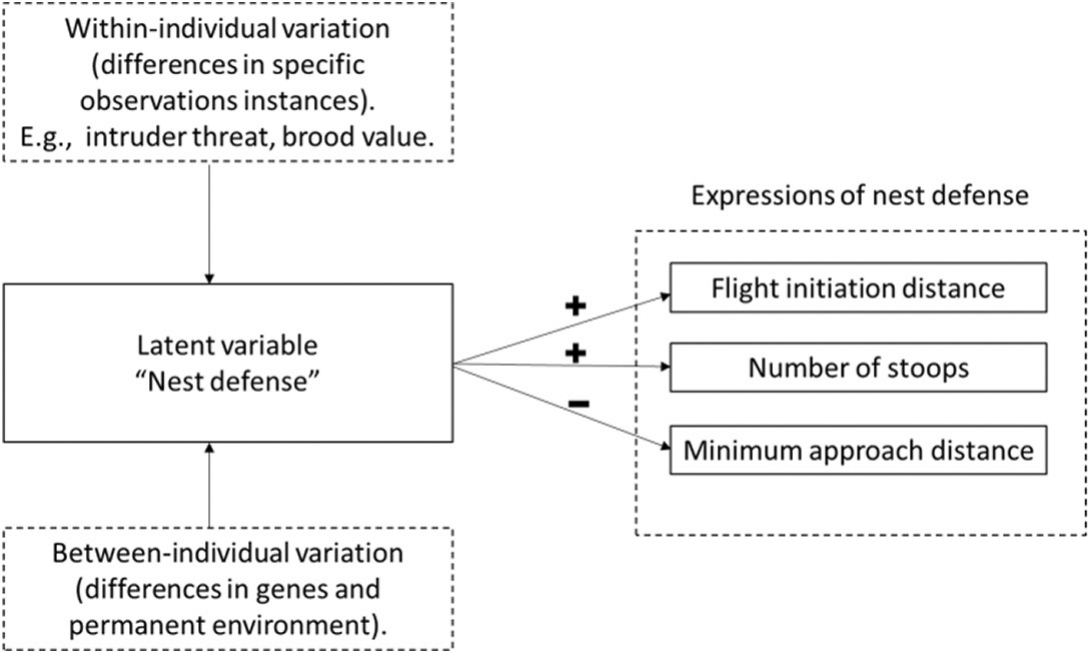
Schematic representation of the behavioral character “nest defense” represented as a latent variable that underlies the expression of observable behaviors including flight initiation distance, number of stoops, and minimum approach distance. Nest defense varies among‐individuals due to genetic and permanent environment effects, but can also vary within‐individuals in response to current environmental conditions. Consequently, correlations between observed behaviors are expected to be quantitatively similar both within‐ and among‐individuals.

We conducted our analyses in several steps. First, we evaluated whether the three different behavioral traits measured during nest defense observations (flight initiation distance in response to an approaching observer (in meters), number of stoops toward the observer, and minimum approach distance to the observer (in meters)) were meaningful expressions of nest defense. If these three behaviors are all expressions of nest defense, then we predicted specific patterns of association between these traits, which could exist both at the within‐ and among‐individual levels as long as the expression of the behaviors vary at those levels (Figure 1). Specifically, we predicted that individuals with long flight initiation distances on average (i.e., indicative of an early initiation of aerial defense) should also stoop toward the observer more often and approach the observer more closely. If the expression of the latent variable is affected by environmental context (e.g., year‐specific environmental conditions, nest stage), then we predict the same patterns of covariation at the within‐individual level.

To test this, we followed the approach used in Araya‐Ajoy and Dingemanse (2014). First, we constructed three separate univariate models (one for each of the behavior traits) using the lme4 package (Bates et al., 2015). We modeled variation in the observed behavior as a function of nest stage (three levels: laying, incubating, provisioning), sex (two levels: male or female), and year (two levels: 2018 or 2019). We did not include approach method (Snowmobile, ATV, boat) because the mode of nest approach changed seasonally and was confounded with nest stage, making it difficult to disentangle these effects. However, these approach methods produced similar levels of disturbance during a nest approach (NAG personal observation), and so are not expected to differentially impact nest defense behavior. Random intercepts were included for the identity of the observer (*n* = 8 levels), and individual id (*N* = 105). FID and minimum approach distance were each log (*x* + 1) transformed and modeled with Gaussian errors, while the number of stoops (untransformed) was modeled with Poisson errors. Adjusted repeatability was calculated as the among‐individual variance divided by the sum of the among‐ and residual variance (Nakagawa & Schielzeth, 2010).

Given the variable effects of nest stage on the different behavior traits (see Results), we next constructed three separate multivariate models to assess whether the expression of each trait correlated across breeding stage contexts (i.e., was a long FID during laying associated with a long FID during incubating and provisioning, etc.). To do this, we constructed separate three trait multivariate models using the MCMCglmm function (Hadfield, 2010) for each behavior (i.e., one for FID, one for Minimum approach distance, and one for number of stoops). Behaviors scored during laying, incubation, and provisioning were treated as separate traits (see Araya‐Ajoy & Dingemanse, 2014). Because observations during one nesting stage by definition could not occur at the same time as observations in other nesting stages, we did not estimate within‐individual correlations between trait pairs. Based on these analyses, we concluded that minimum approach distance and number of stoops were both expressions of nest defense, but that FID was shaped by additional constraints (see Results). Thus, as a final step, we constructed a bivariate model for minimum approach distance and number of stoops using the MCMglmm function (Hadfield, 2010) to evaluate their within‐ and among‐individual covariance. If both are expressions of nest defense, we would expect them to exhibit positive covariance both within‐ and among‐individuals.

The analyses described above served as an exploratory tool that helped determine which measure of nest defense would be used for subsequent analysis (Dingemanse & Wright, 2020; Dochtermann & Nelson, 2014). Although our analyses indicate that both minimum approach distance and number of stoops are expression of nest defense, we used minimum approach distance as our measure of nest defense in our analyses because it offered two advantages over number of stoops. First, it is a continuous trait that could be modeled with gaussian errors, allowing us to estimate within‐individual variance. Second, we achieved better model convergence in models with approach distance. However, we did run all models using number of stoops to confirm that our choice of nest defense proxy did not unduly influence our results, which it did not (results not shown). Further, because analyses in which minimum approach distances >100 m were excluded from the data set yielded quantitatively similar results to analyses in which minimum approach distances >100 m were truncated to 100 m, we chose to use the latter in the analyses presented in the main text as it increased the number of useable observations from *N* = 351 to *N* = 369.

Next, we used the R package lme4 (Bates et al., 2015) to model variation in nest defense (minimum approach distance to observer) to assess state dependence and estimate short‐ and long‐term repeatability of nest defense behavior (Araya‐Ajoy et al., 2015). Minimum approach distance was log(*x* + 1) transformed prior to analyses to meet model assumptions of normally distributed residuals. “Nest Stage” varies within a season, and served as our proxy for brood value. Note, because we were interested in estimating the extent to which individuals exhibit repeatable differences in nest defense, we did not model individual characteristics (other than sex) that might co‐vary with nest defense (e.g., brood size or lay date), as this would have resulted in an underestimate of the repeatability of nest defense. We predicted an increase in nest defense behavior with increasing nest stage. We also expected females to respond more strongly than males to increasing nest stage, and consequently, we included an interaction between “Nest Stage” and “Sex.” We included random effects of “ID” and “ID_Series.” “ID” represented an individual peregrine and their corresponding color‐coded band number (*N* = 32 individuals) or in cases in which an individual was not banded we used their NestID (random ID given to each nest every year) and sex of the individual (*N* = 64 individuals). We recognize that using this approach, unmarked individuals may be assigned as unique individuals in each year of the study. This would tend to underestimate among‐individual variance, and is thus conservative given our objective of estimating repeatability of nest defense. “ID Series” represented an individual's “ID” plus the year of the study to form a series. This allowed us to calculate short‐term repeatability using the formula (*V*_individual_ + *V*_individual_series_)/(*V*_individual_ + *V*_individual_series_ + *V*_residual_), and long‐term repeatability using the formula (*V*_individual_)/(*V*_individual_ + *V*_individual_series_ + *V*_residual_; Araya‐Ajoy et al., 2015). Where *V*_individual_ is the variance explained by the individual, *V*_individual_series_ is the variance explained by the series, and *V*_residual_ is the residual variance (Araya‐Ajoy et al., 2015; Nakagawa & Schielzeth, 2010). We calculated repeatability using the formula (*V*_individual_)/(*V*_individual_ + *V*_residual_) for each measure of nest defense (Dingemanse & Dochtermann, 2013; Nakagawa & Schielzeth, 2010). Where *V*_individual_ is the variance explained by individual ID and *V*_residual_ is the residual variance. We assessed model fit by visual inspection of fitted versus residual plots. We then used the “sim” function from the “arm” package to generate 1000 simulations of the posterior distribution of the model parameters (Gelman & Su, 2015). We used the package “MCMCglmm” to extract 95% credible intervals (CrI) around the mode (β) of the estimated effect using the 1000 simulations of the model, which represents the uncertainty in our measurements (Hadfield, 2010).

Finally, we used the R package MCMCglmm and constructed multivariate models (generalized linear mixed‐effect model using Markov Chain Monte Carlo simulations) to estimate (dis‐)assortative mating and selection gradients (Hadfield, 2010; Houslay & Wilson, 2017). We fitted male nest defense (log minimum approach distance in meters), and female nest defense (log minimum approach distance in meters) as response variables. We multiplied this by −1 prior to analyses so that smaller values were indicative of lower nest defense behavior. This made interpretation of selection gradients more intuitive. Positive selection corresponded to selection for higher nest defense, and negative selection corresponded to selection for lower nest defense. Both male and female nest defense included repeated measures within each year, as such, we could estimate within‐pair correlation between male and female nest defense as a way of evaluating the effect of shared labile environment (Hadfield, 2010) on nest defense behavior in addition to the among‐pair correlation (i.e., shared nonlabile environment and/or (dis‐)assortative mating; Class et al., 2017). Utilizing all nest defense tests instead of producing an average phenotypic score has been demonstrated to produce more accurate estimates of selection gradients (Dingemanse et al., 2021).

Because fluctuation selection and nonrandom mating are not mutually exclusive mechanisms and may have interactive effects, we fitted a model for each year of the study (i.e., 2018, 2019) separately, as well as a model combining both years. For each of these models, we used a three‐trait prior specification that could account for one random effect (Nest ID). In our model that combined years, we created a series that combined Nest ID and study year (i.e., 45_2018) as a random effect. We had incomplete banding recordings (see above) and could not identify pair turnover between years with certainty. We calculated relative fitness by taking the number of nestlings fledged for a given nest and dividing it by the population mean number of nestlings fledged in that year. The residual variance for fitness was set close to zero (0.0001) since fitness was only measured once per season for each individual, and we did not estimate within‐individual covariance that included relative fitness (see Houslay & Wilson, 2017). Each trait was modeled with gaussian errors. We performed 1,300,000 iterations of each model with a thinning interval of 1000 and a burn‐in of 300,000 to achieve an effective sample size of 1000 simulations. We assessed model fit visually be inspecting trace and density plots and we checked for autocorrelation using the function *autocorr.diag* (for model diagnostic tests and plots for all models see https://github.com/nuwcru/Rankin_Inlet‐Nest_Defense_PEFA).

For all analyses, we used the mode (β) of estimated effects and 95% CrI to evaluate support for each effect. We describe effects and associated CrI that do not overlap zero as showing strong support for an effect. CrIs that were centered around zero (i.e., equal distribution of CrI on both sides of zero) with an estimated effect near zero were interpreted as showing “no support” or “strong support for no effect.” CrIs that overlapped with zero but were not centered around zero (≤15% overlap) were interpreted as showing “moderate support” for an effect since a posterior distribution that overlaps zero by ≤0.15 corresponds to more than five times greater support for an interpretation of an effect in the estimated direction than it does for an effect in the opposite direction (Marsman & Wagenmakers, 2017). For contrasts between pairs of independent estimates, we computed the difference between the two posterior distributions, and interpret support for a difference as above (i.e., strong support = 95% CrI of estimated difference does not overlap zero, moderate support = 95% CrI of estimated difference overlaps zero by ≤0.15, and no support for a difference = 95% CrI of estimated difference overlaps zero by >0.15). Random effects are constrained to be positive, thus, random effects whose estimate or lower 95% CrI approached zero were interpreted as lacking support.

## 3 RESULTS

### 3.1 Evaluating alternative measures of nest defense

Each of the three behaviors scored during nest defense observations harbored significant repeatable among‐individual variation (Table 1). Further, each of these traits exhibited within‐individual plasticity in response to breeding context and year (Table 1). However, the effects of breeding context were variable across traits. Number of stoops increased and minimum approach distance decreased with progressing nest stage, indicative of increasing nest defense levels with increasing brood value. FID was affected by nest stage, but in a different way compared to minimum approach distance or number of stoops. FID decreased during incubation, which would be interpreted as lower nest defense. However, this may also reflect fundamentally different costs and benefits of leaving the nest during incubation compared with other nest stages. Observer identity explained little, if any, variation in the traits (Table 1).

**TABLE 1:** Sources of variation in flight initiation distance, minimum approach distance, and number to stoops

	FID (m)^a^	Minimum approach distance (m)^b^ distances >100 m excluded	Minimum approach distance (m)^b^ distances truncated to 100 m	Number of stoops
**Fixed effects**	**β (95% CrI)**	**β (95% CrI)**	**β (95% CrI)**	**β (95% CrI)**
Intercept^c^	4.49 (4.12, 4.76)	2.94 (2.77, 3.30)	2.90 (2.68, 3.31)	0.60 (0.12, 1.11)
Sex	0.48 (0.11, 0.73)	−0.26 (−0.61, −0.10)	−0.11 (−0.46, 0.14)	0.75 (0.09, 1.01)
Nest stage				
Incubation	−0.49 (−0.72, −0.16)	−0.25 (−0.48, 0.00)	−0.36 (−0.57, −0.12)	0.24 (0.02, 0.38)
Provisioning	−0.10 (−0.37, 0.21)	−0.45 (−0.81, −0.34)	−0.54 (−0.84, −0.36)	0.18 (0.05, 0.41)
Year (2019)	−0.36 (−0.55, −0.01)	0.05 (−0.19, 0.25)	0.29 (−0.14, 0.43)	−0.29 (−0.51, −0.12)
**Random effects**	**σ** ^**2**^ **(95% CrI)**	**σ** ^**2**^ **(95% CrI)**	**σ** ^**2**^ **(95% CrI)**	**σ** ^**2**^ **(95% CrI)**
ID	0.21 (0.15, 0.26)	0.26 (0.25, 0.39)	0.31 (0.27, 0.42)	1.32 (1.21, 1.59)
Observer	0.00	0.02 (0.01, 0.05)	0.07, 0.02, 0.12)	0.31 (0.23, 0.42)
Residual	0.52 (0.47, 0.63)	0.52 (0.47, 0.63)	0.61 (0.51, 0.69)	1
**Repeatability**	***r* (95% CrI)**	***r* (95% CrI)**	***r* (95% CrI)**	***r* (95% CrI)**
ID	0.27 (0.21, 0.35)	0.36 (0.30, 0.41)	0.36 (0.31, 0.40)	0.57 (0.55, 0.62)

^a^ Ln (FID + 1) transformed.

^b^ Ln (minimum approach distance + 1) transformed.

^c^ Intercept is for reference categories female, egg‐laying, year 2018.

Results from multivariate analyses corroborated the interpretations from the univariate analyses described above (Table 2). Minimum approach distance and number of stoops were both positively correlated across breeding contexts as expected if they are expressions of the same trait. However, this was not the case for FID, which showed variable (null, negative, or positive) cross‐context correlations (Table 2). Again, this is consistent with the interpretation that variation in FID is an expression of more than just nest defense (e.g., incubation behavior), and that the relative contribution of each to the expression of FID varies with nest stage.

**TABLE 2:** Across context correlations for each of the three behavioral measures: (A) Flight initiation distance, (B) Minimum approach distance, and (C) Number of stoops

(A)	FID (laying)	FID (incubating)	FID (provisioning
FID (laying)	‐	−0.01 (−0.50, 0.45)	−0.35 (−0.73, 0.18)
FID (incubating)		‐	0.31 (−0.10, 0.66)
FID (provisioning)			‐

(B)	Min (laying)	Min (incubating)	Min (provisioning
Min (laying)	‐	0.28 (−0.18, 0.61)	0.23 (−0.24, 0.64)
Min (incubating)		‐	0.46 (0.12, 0.74)
Min (provisioning)			‐

(C)	Stoops (laying)	Stoops (incubating)	Stoops (provisioning
Stoops (laying)	‐	0.55 (0.07, 0.84)	0.66 (0.22, 0.89)
Stoops (incubating)		‐	0.76 (0.50, 0.91)
Stoops (provisioning)			‐

*Note*: Among‐individual correlations are presented above the diagonal. Within‐individual correlations were not estimated because by definition, a behavior expressed during one nest stage could not simultaneously be expressed in another nest stage by the same individual.

Based on these analyses, we conclude that both minimum approach distance and number of stoops are expressions of nest defense during egg laying, incubation, and provisioning nest stages. A bivariate model of these two traits further corroborated this interpretation, as minimum distance and number of stoops show the same covariance both within‐ (*r* = −0.46, 95% CrI = −0.57, −0.35) and between‐individuals (*r* = −0.75, 95% CrI = −0.89, −0.54). Both within and between individuals, more stoops were associated with closer approach distances. This result was nearly identical if we used a larger data set in which minimum approach distances >100 m were truncated to 100 (*N* = 369 observations): within individual correlation (*r* = −0.53, 95% CrI = −0.63, −0.43) and among‐individual correlation (*r* = −0.75, 95% CrI = −0.90, −0.54).

### 3.2 State‐dependence and repeatability of nest defense

**FIGURE 2:**
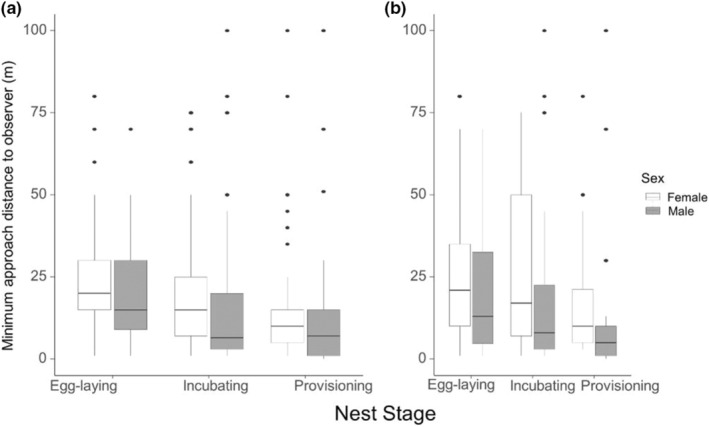
Nest defense, scored as the minimum approach distance to the observer as a function of nest stage and sex (females in red, males in blue) and year (panel A = 2018, panel B = 2019). Shorter approach distances are interpreted as higher nest defense. Box plots illustrate raw data for minimum approach distances (m). Lines within the boxes mark the medians, boxes span the 25th and 75th interquartile range, and the whiskers indicate the 90th and 10th percentiles.

We conducted a total of 227 nest visits to score nest defense. Of these, there were 213 cases where at least one parent was present, for a total of 369 nest defense tests (218 female, 151 male). In 2018, 55 individuals were tested (29 females, 26 males), and in 2019, 62 individuals were tested (34 females, 28 males). There were 14 individuals (10 females, four males) that were tested in both seasons. In 2018, there was an average of four tests per female and three tests per male, and in 2019, an average of three tests per female and three tests per male. Analyses of log‐transformed nest defense as a function of “Sex,” “Nest Stage,” and “Year” found varying degrees support for effects of sex, nest stage, and year on nest defense (Table 3). There was no support for an interaction between sex and nest stage (Table 3). However, on average, males tended to approach more closely compared with females (β = −0.15, 95% CrI = −0.50, 0.34), indicative of higher nest defense. However, the difference between males and females was not significant (95% CrI overlapped zero). Minimum approach distances differed significantly as a function of nest stage (Table 3). Minimum approach distances were highest during egg laying, and lowest during provisioning, indicative of a progressive increase in nest defense across nesting stages (Figure 2). We found strong support for an effect of “Year” (β = 0.28, 95% CrI = 0.08, 0.56) with shorter approach distances in 2018 compared to 2019. We found strong support for moderate short‐term repeatability (i.e., within‐year; *r* = 0.37, 95% CrI = 0.32 0.42) and long‐term repeatability (i.e., between‐year) repeatability (*r* = 0.30, 95% CrI = 0.24, 0.35) of nest defense behavior. As expected, repeatability decreased with increasing time interval.

**TABLE 3:** Univariate analyses of sources of variation in nest defense (log‐minimum distance to observer (m) + 1)

	Nest defense log(minimum distance to observer (m) + 1)
**Fixed effects**	**β (95% CI)**
Intercept^a^	2.91 (2.63, 3.23)
Sex (Male)	−0.15 (−0.50, 0.34)
Nest stage	
Incubation	−0.34 (−0.61, 0.03)
Provisioning	−0.60 (−0.83, −0.23)
Sex: nest stage	
Male: incubation	−0.12 (−0.57, 0.41)
Male: provisioning	−0.25 (−0.57, 0.41)
Year^b^	0.28 (0.08, 0.56)
**Random effects**	**σ (95% CrI)**
Individual	0.29 (0.23, 0.40)
Individual series	0.07 (0.05, 0.09)
Residual	0.66 (0.56, 0.75)
**Repeatability**	***r* (95% CrI)**
Short‐term	0.37 (0.32, 0.42)
Long‐term	0.30 (0.24, 0.35)

*Note*: Adjusted short‐ and long‐term repeatability were calculated following Araya‐Ajoy et al. (2015). The posterior mode (β), and 95% credible intervals (CrI) are reported.

a Intercept represents female nest defense during egg‐laying in the first year (2018).

b Year represents the estimate for the second year (2019).

### 3.3 (Dis‐)assortative mating and selection gradients

**FIGURE 3:**
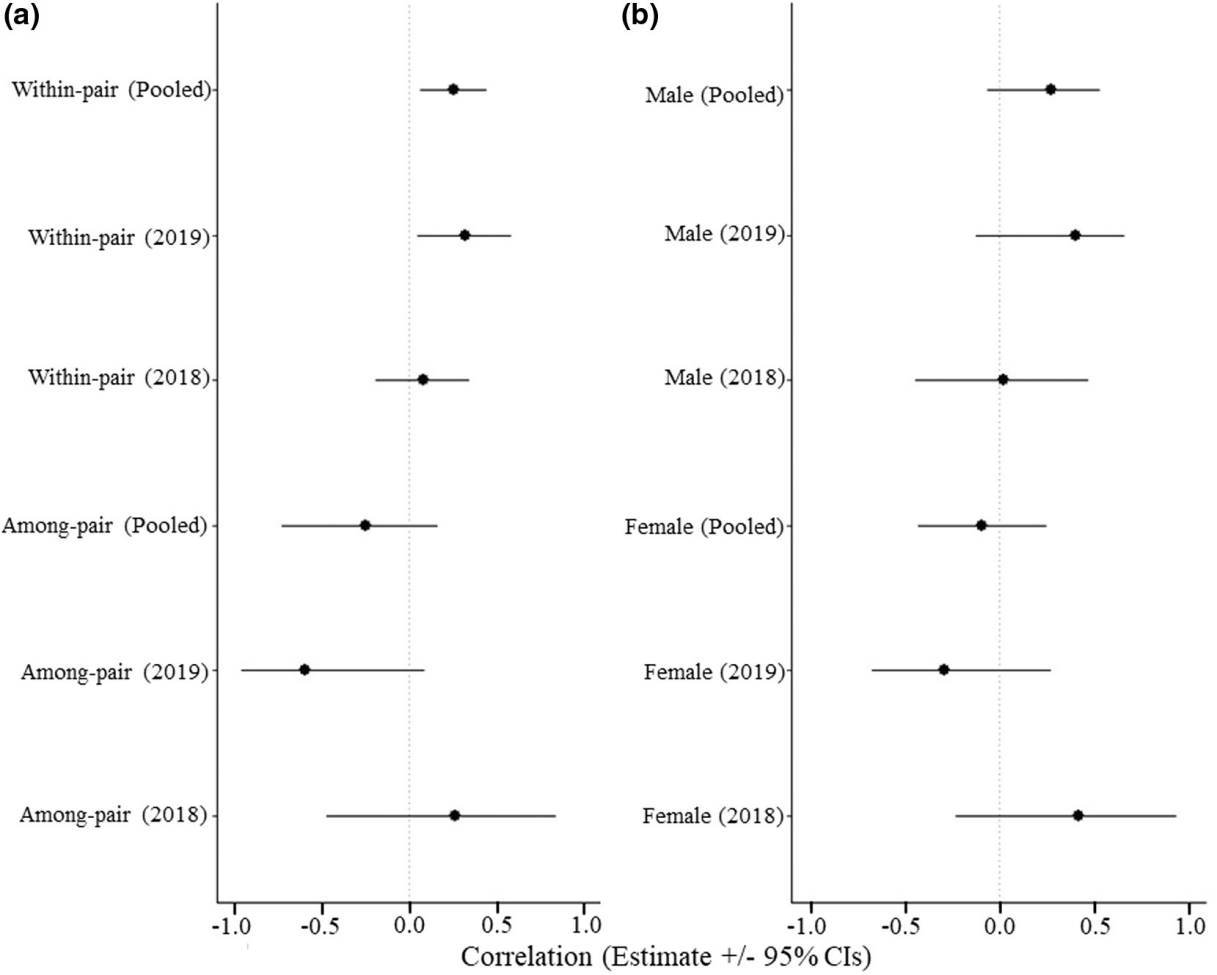
(a) Within‐ and among‐pair correlations for nest defense behavior in 2018, 2019, and both years pooled, and (b) standardized selection coefficients for nest defense behavior for each combination of sex and year, and both years pooled. Nest defense was scored as minimum approach distance to observer, log(*x* + 1) transformed, and multiplied by −1 prior to analyses so that positive selection estimates correspond to selection for greater nest defense, and negative estimates correspond to selection for lower nest defense. Points represent mean estimated correlation coefficient, and whiskers denote 95% CrIs.

We had 129 unique pair‐level tests (2018 = 70 tests, 2019 = 59 tests) at 57 nests (2018 = 25 nests, 2019 = 26 nests) for which both the male and female were present during nest defense observations. Among‐pair correlations tended to be negative overall when years were pooled (*r* = −0.31, 95% CrI = −0.74, 0.12). However, the among‐pair correlations were qualitatively different across our two study years (Figure 3a). In 2018, the estimated among‐pair correlation was positive, but the 95% CrI overlapped zero substantially (*r* = 0.21, 95% CrI = −0.40, 0.84), while in 2019, the estimated among‐pair correlation for nest defense was negative (*r* = −0.53, 95% CrI = −0.97, 0.04), with less than 6.2% of estimates being positive. There was marginal support that these year effects differed (β = 0.63, 95% CrI = −0.28, 1.46, proportion overlap across year = 0.08).

Within‐pairs, nest defense scores tended to be positively correlated in both years, however the correlation was significant in 2019 (*r* = 0.38, 95% CrI = 0.05, 0.60), but not in 2018 (*r* = 0.14, 95% CrI = −0.17, 0.36; Figure 3a). Computing the difference between the posterior distributions for each year indicated that there was moderate support that the correlation was stronger in 2019 (β = 0.34, 9% CrI = −0.15, 0.61, proportion of estimates overlapping zero = 0.12). When years were pooled there was strong support for a positive within‐pair correlation (*r* = 0.23, 95% CrI = 0.04, 0.43).

Selection on nest defense behavior varied across sexes and years (Figure 3b). The point estimate for selection on male nest defense behavior was positive in both years (2018, β = 0.05, 95% CrI = −0.41, 0.48; 2019: β = 0.33, 95% CrI = −0.15, 0.65), such that males with higher nest defense scores (i.e., shorter approach distances) tended to have higher fitness. Although support for selection on nest defense was stronger in 2019, in both years the estimates overlapped zero. Pooling the selection estimates across both years resulted in moderate support for positive selection on male nest (β = 0.26, 95% CrI = −0.08, 0.53) suggesting that the year‐specific analyses may have lacked power. The proportion of estimates overlapping zero was *p* = .07.

The year‐specific patterns of selection on nest defense in females were different from in males. In 2018, selection on female nest defense tended to be positive (β = 0.25, 96% CrI = −0.24, 0.82), while in 2019, selection on female nest defense tended to be negative (β = −0.30, 95% CrI = −0.70, 0.21). Overall, there was moderate support that selection on female nest defense differed across our two study years (β = 0.55, 95% CrI = −0.18, 1.28, proportion of estimates overlapping zero = 0.09; Figure 3b). Consequently, when years were pooled there was no support for selection on female nest defense (β = −0.10, 95% CrI = −0.45, 0.27).

## 4 DISCUSSION

We evaluated support for three mechanisms that have been proposed to contribute to the maintenance of among‐individual differences in nest defense behavior: state‐dependence, assortative mating, and fluctuating selection. We found that peregrines adjusted their nest defense in response to nesting stage and year, revealing plastic, state‐dependent, adjustment of nest defense. At the same time, nest defense behavior was repeatable in peregrine falcons both within and between years. We found some evidence for assortative mating and fitness consequences, but these relationships differed between the two study years. We discuss these results in light of how assortative mating versus plastic adjustment of nest defense might allow peregrines to respond to temporal variation in the environment that shape the benefits of nest defense.

We found support for plastic, within‐individual adjustment in nest defense. Specifically, nest defense increased (i.e., individuals stooped closer to the observer on average) as a function of nest stage with the closest distances occurring during the provisioning stage and the furthest distances during egg laying (Table 3, Figure 2). We also found support for positive within‐pair correlations between male nest defense and female nest defense in both study years and when combining years, indicating that males and females are adjusting their investment in nest defense similarly in response to their shared labile environment. An increase in nest defense with nest stage progression occurred in both sexes and in both study years, and is in line with numerous other empirical studies that similarly document increasing nest defense as nest stage progresses (reviewed in Knight & Temple, 1986). This pattern is generally thought to reflect adaptive parental investment (i.e., increased investment in nest defense with increasing brood value; Montgomerie & Weatherhead, 1988; Trivers, 1972). The breeding season in the Arctic is a short window and there is low opportunity for re‐nesting once a pair has progressed significantly into the breeding season (Bradley et al., 1997; Falk et al., 1986). Peregrines breeding in Rankin Inlet have a narrow range of laying dates to successfully produce nestlings before the seasonal decline in resources (~12 days, Bradley et al., 1997). Increased nest defense over the nesting stage likely reflects the increased chance of fledging a nestling and simultaneously the low opportunity for re‐nesting. Another possibility is that the increasing nest defense with progressing nest stage was not due to increased brood value, but instead, reflected a shift in nest defense strategy. For example, during laying and incubation, peregrines may rely primarily on crypsis for nest defense, and shift to more to active nest defense when nests become conspicuous due to regular provisioning visits by parents and begging by nestlings. We suggest this is unlikely given that aggressive territory defense occurred at all stages, including prior to egg laying.

Moreover, while peregrines exhibited plastic adjustment of nest defense as a function of nest stage and year (Figure 1), they also showed repeatable variation in nest defense over both the short‐ (within‐year repeatability = 0.38) and long‐term (across‐year repeatability = 0.30), controlling for sex. Individuals that tended to have close approach distance in 1 year, or one nest stage, tended to have close approach distances in the other year and in other nest stages. Similar repeatability of nest defense has been reported in at least three other raptor species (Montagu's harrier: Arroyo et al., 2017; Ural owls: Kontiainen et al., 2009; goshawk: Møller & Nielsen, 2014).

Many raptors have reverse sexual size dimorphism, with females being larger than males. In these species, females often invest more in nest defense compared with males (e.g., Arroyo et al., 2017; Kontiainen et al., 2009; Møller & Nielsen, 2014). Because peregrines are also reverse sexually size dimorphic (White et al., 2002), we considered sex as a potential state‐variable shaping nest defense behavior. Surprisingly, we found no evidence for sex‐related differences in the strength of nest defense as measured by the minimum approach distance to an observer (Table 3, Figure 2). However, posthoc analyses revealed that of the 369 nest defense observations made in our study, 218 were of females and 151 were of males. This is significantly different from a null expectation of equal probability of nest defense response for males and females (χ^2^ = 6.125, *p* = .01), indicating that females were significantly more likely to be present in the vicinity of the nest and engage in nest defense behavior compared with males. Thus, while males and females have similar levels of nest defense when nest defense is expressed, females are more likely to express nest defense behavior compared with males.

Given that peregrines exhibit repeatable among‐individual differences in nest defense, we also evaluated support for (dis)assortative mating and/or selection on nest defense as potential mechanisms maintaining among‐individual variation in nest defense in this population. In both years combined (2018 and 2019), there was moderate support for disassortative mating, with positive selection for male nest defense and no selection for female nest defense. However, our year‐specific analyses suggest that patterns of nonrandom mating and selection on nest defense likely differed across years. In the first study year (2018), there was no evidence for assortative mating, but moderate support for positive selection on female nest defense with no evidence for selection on male nest defense. In contrast, in the second year of the study (2019), there was moderate support for disassortative mating, with selection against female nest defense, and selection for male nest defense. The finding that patterns of (dis‐)assortative mating may have differed across years has two important implications. First, it reveals that the observed patterns of assortative mating cannot be due to shared response to particular combinations of observers (Wang et al., 2019), as this would result in similar patterns in both years. This is consistent with findings from univariate analyses showing that observer ID explained almost no variance in nest defense behavior. Second, it indicates that the observed patterns cannot be explained by shared environment effects alone, as in that case, we would expect to observe positive assortative mating in both years (Class & Brommer, 2018).

Although the estimated effect sizes include substantial uncertainty, our results are suggestive of differences in patterns of assortative mating across our two study years. One interpretation for these results is that fluctuating selection on male and/or female nest defense resulted in changing mate choice decisions across years. In the first year, when there was no selection on male nest defense, females did not select males on the basis of the nest defense phenotypes. In the second year, when low female nest defense and/or high male nest defense were selected for, females with low nest defense preferentially mated with high nest defense males. We suggest that this interpretation is unlikely because adaptive changes in mate choice across years would require that peregrines are (1) able to evaluate early in the season which behavioral type(s) would be selected for in that year and (2) determine the nest defense phenotype of potential partners before nest defense behavior is expressed. Although the latter is possible if nest defense correlates with other phenotypic characters, the former is less likely. Given that peregrines arrive in Rankin Inlet when there can still be significant snow cover, it seems unlikely that they would be able to evaluate which phenotype would be favored later in the season.

We propose two alternative nonexclusive explanations for the observed disassortative mating in 2019. First, these patterns may reflect plastic adjustment by males and/or females to current environmental conditions, including the behavioral type of their partner. Although nest defense was repeatable both within‐ and across years, the observed inter‐annual repeatability of circa 0.30 still leaves substantial scope for plastic adjustment (Dingemanse & Dochtermann, 2013). In 2019, conditions were more challenging; temperatures were lower, and there were more heavy rain events (NAG, personal observation), such that high nest defense may have been selected against if high nest defense comes at the cost of other parental investment behaviors, such as provisioning (e.g., Mutzel, Blom, et al., 2013). Consistent with this view, both males and females showed lower nest defense (i.e., longer minimum approach distances) on average in 2019. However, to generate the pattern of disassortative mating observed in this year would necessitate that male and female plasticity were inversely related. In other words, in pairs where females exhibited small reductions in nest defense, males exhibited large reductions, and vice versa. This would result in a negative correlation between male and female nest defense within breeding pairs. Further, the negative correlation between male and female nest defense means that the observed sex‐specific selection on nest defense may be due to the behavioral type of either, or both, parent(s). Unfortunately, in this study, the number of banded individuals that were observed in both years of the study was too small to allow us to allow us to directly test whether male and female interannual plasticity were negatively correlated (*N* = 4 males, *N* = 10 females).

This posthoc interpretation assumes that nest defense is negatively correlated with other forms of parental care such as provisioning. Although some studies have reported negative correlations between nest defense and provisioning (Mutzel, Blom, et al., 2013; Mutzel, Dingemanse, et al., 2013), others have reported positive (Rytkönen et al., 1995), or no correlations (Kontiainen et al., 2009), indicating that the pattern is variable both across species and across sexes within the same species. When pooling both years, we found moderate support for the interpretation that male nest defense is selected for suggesting that defensive behavioral types might (i) be of higher quality, (2) able to invest in high levels of both nest defense and provisioning without a cost, and/or (3) able to adjust to environmental conditions more quickly than less defensive behavioral types (Betini & Norris, 2012). Thus, assessment of the sex‐specific relationships between nest defense and provisioning effort in peregrines are needed. Unlike in some species, where the parents deliver food to the young that they themselves acquired, in peregrines, females typically provision food to the young even if the prey was captured by the male of the pair (White et al., 2002). Thus, observations of prey deliveries alone may be insufficient to tease apart sex‐specific provisioning effort, and data on individual foraging trips (e.g., collected using GPS tags on individuals) may be needed to disentangle male and female provisioning effort.

Another possibility is that high turnover at nesting sites due to high adult mortality between our two study years could have generated the observed disassortative mating pattern in 2019. Peregrines generally exhibit high site fidelity across years (>70%; Court et al., 1989), and in many species, nest defense increases with parental age (Pearson et al., 2005). These two factors combined mean that on average, we would expect positive assortative mating by age, with older, more defensive birds being paired to other older, more defense birds. If there was high annual mortality between the 2018 and 2019 breeding seasons, this could have resulted in a larger fraction of pairs being mated disassortatively by age in the second year. In this case, the negative effect of female nest defense on fledging success may be due to older females with high nest defensiveness being mated with younger, less experienced males, rather than a direct effect of nest defense per se. This is speculative, and the incomplete banding records in our study population preclude us from testing this idea directly. However, at least one other study has reported that patterns of disassortative mating are age‐dependent (Dingemanse et al., 2004).

## 5 CONCLUSION

Here, we show that nest defense behavior in Arctic breeding peregrines is both plastic and repeatable. Peregrines increased nest defense as the breeding season progressed, and also adjusted intensity of nest defense across years, presumably in response to year‐specific conditions. We found some support for fluctuating selection as a potential mechanism maintaining variation in nest defense in the population. We also observed year‐specific patterns of assortative mating; however, our data do not allow us to differentiate between multiple mechanisms that could have generated these patterns. Future work is needed that tracks a larger number of marked individuals over more than 2 years to allow for direct evaluation of the relative role of inter‐annual plasticity versus changes in population age structure in generating changes in patterns of assortative mating.

## AUTHOR CONTRIBUTIONS

**Nick Gulotta:** Conceptualization (equal); data curation (lead); formal analysis (lead); funding acquisition (supporting); investigation (lead); visualization (lead); writing – original draft (lead); writing – review and editing (equal). **Kimberley Mathot:** Conceptualization (equal); formal analysis (supporting); funding acquisition (lead); supervision (lead); visualization (supporting); writing – original draft (supporting); writing – review and editing (equal).

## FUNDING INFORMATION

This work was supported through NSERC grants to KJM (NSERC Discovery Grant #RGPIN‐2018‐04358, NSERC Northern Research Supplement: NRS‐2018‐517979), a Mitacs Accelerate Internship to NG (grant #IT18033) sponsored by Alastair Franke, government of Nunavut funding held by Alastair Franke (SC180029, SC180030, SC190039, SC190042), Nunavut General Monitoring Program funding held by Alastair Franke (EC73_2019–20), UAlberta North (held by NG), and in‐kind funding to Alastair Franke from Nunavut Arctic College and Agnico Eagle Mines (Meliadine Division).

### OPEN RESEARCH BADGES

This article has earned Open Data and Open Materials badges. Data and materials are available at https://github.com/nuwcru/Rankin_Inlet‐Nest_Defense_PEFA.

## Data Availability

All R‐scripts and data required to replicate the analyses presented in this manuscript are available on GitHub (https://github.com/nuwcru/Rankin_Inlet‐Nest_Defense_PEFA).
